# Depressive Symptoms, Dietary Patterns and Other Factors Associated with Constipation in Middle-Aged Adults from Kielce District in Poland: A Cross-Sectional Study

**DOI:** 10.3390/jcm14197090

**Published:** 2025-10-08

**Authors:** Martyna Głuszek-Osuch, Elżbieta Cieśla, Kamila Sobaś, Edyta Suliga, Stanisław Głuszek

**Affiliations:** 1 Institute of Health Sciences, Collegium Medicum, Jan Kochanowski University in Kielce, 25-369 Kielce, Poland; martynaosuch1@gmail.com (M.G.-O.); eciesla@ujk.edu.pl (E.C.); ksobas@ujk.edu.pl (K.S.); edyta.suliga@ujk.edu.pl (E.S.); 2 Institute of Genetics and Animal Biotechnology, Polish Academy of Sciences, 05-552 Magdalenka, Poland; 3 Department of Surgical Oncology, Holy Cross Cancer Centre in Kielce, 25-734 Kielce, Poland; 4 Department of Surgery, Hospital of the Ministry of Interior and Administration in Kielce, 25-375 Kielce, Poland

**Keywords:** stool frequency, depressive symptoms, dietary patterns, sitting time

## Abstract

**Background:** Many factors associated with constipation have not been sufficiently investigated. The aim of this study was to assess the factors associated with constipation, defined as low stool frequency, in a community-dwelling adult population. **Methods:** A cross-sectional study was carried out between 2010 and 2012 among adults who live in the Kielce District of Poland. The participants comprised 11,488 persons aged 37–66 years. Data on depressive symptoms, eating frequency, physical activity and smoking, as well as sociodemographic data, were collected using face-to-face interviews. Constipation was defined as < 3 defecations per week. Dietary patterns (DPs) were identified using a factor analysis. Associations between DPs and the likelihood of constipation were assessed using a logistic regression analysis. **Results:** The factors associated with constipation were moderate and severe depressive symptoms, regular use of antidepressants, a sedentary lifestyle and past smoking (in women) (all *p* < 0.05). The likelihood of constipation was significantly decreased by male sex and obesity, as well as by a diet belonging to the highest terciles of the ‘Carbohydrates’, ‘Vegetables and Meat’ and ‘Healthy’ DPs (all *p* < 0.05). For the ‘Unhealthy’ DP, a high level of physical activity (Q4 vs. Q1 of MVPA) decreased the likelihood of constipation (OR = 0.75; 95%CI: 0.57–0.99). **Conclusions:** Preventing the onset or reoccurrence of depression by improving one’s mental resistance, reducing the risk factors for depression and promoting a healthy lifestyle, primarily a diet high in vegetables and a limited sitting time, reduces the risk of constipation. Further longitudinal studies are needed to better understand which factors are associated with the occurrence of constipation.

## 1. Introduction

A major problem in the functioning of the gastrointestinal tract is peristalsis disorders, i.e., any abnormality related to the movement of food through the gastrointestinal tract [1]. Symptoms of peristalsis disorders include diarrhoea, stomach pain and flatulence. Reduced peristalsis leads to constipation. This problem is experienced by a considerable portion of the adult human population throughout the world. The prevalence of constipation in the general population ranges from 0.7% to 79.0%, depending on the diagnostic criteria and the patient’s age [2,3]. A typical, and the most commonly used, indicator of constipation is low stool frequency, with at least three defecations per week being considered the norm [4]. Constipation is a problem that affects not only an individual’s physical health, but also their emotional well-being and overall quality of life [5]. The causes of constipation are complex. It may result from a bad diet, sedentary lifestyle, demographic factors, genetic predispositions, metabolic and hormonal disorders, psychological states, gastrointestinal diseases (cancer, inflammations that reduce the intestinal lumen or peritoneal adhesion disease) and the use of some medications [2,5,6]. A higher prevalence of constipation is observed in women than in men [2,7], as well as in elderly persons [2,8]. Most studies to date have focused on the effects of individual nutrients or food products on the incidence of constipation [9,10,11]. Systematic reviews and meta-analyses of randomised controlled trials showed that a high intake of fruits, vegetables, beans and whole grains, which are the main source of fibre, and magnesium oxide supplements plays a key role in ensuring regular defecation and preventing constipation [10,11]. However, DPs are a much better indicator of a person’s real dietary habits. Dietary patterns are complex variables that cover the intake of many different food groups and take into account the interactions between nutrients [12]. At the time of writing this article, few studies have been conducted on the relationship between DPs and the risk of constipation [13,14,15], and their results are specific to the parts of the world that they were conducted in (Asia and the Middle East) and the dietary habits characteristic of those populations, which limits their generalisability over other populations. The results of these cross-sectional studies suggested that following traditional DPs in those regions reduced the risk of constipation, whereas an unhealthy Western diet increased it [13,14,15]. The latest prospective analysis of DPs, conducted in three cohorts of middle-aged and older adults (US), confirmed that long-term adherence to a Western diet increased the risk of constipation, whereas adherence to a Mediterranean diet reduced it [16]. These associations were independent of the intake of total fibre and ultra-processed foods.

Some researchers have suggested that the risk of constipation is lower in current smokers, which may be due to a reduced intestinal transit time in smokers [17]. However, other studies have demonstrated that smoking is not associated with constipation, stool frequency or stool texture [18,19]. On the other hand, quitting smoking, despite its long-term health benefits, may be associated with certain harmful side effects, including constipation [20,21,22]. The results of three cycles of research by the National Health and Nutrition Examination Survey (NHANES) in the US have shown that obesity and constipation share common risk factors [23]. Nonetheless, few studies concerning the associations between obesity and an increased risk of constipation have been conducted, and their results have been ambiguous [24,25,26]. However, a lack of physical activity (PA) was associated with a high frequency of constipation [7].

The results of the studies conducted in many countries have demonstrated that persons with depression are at a much higher risk of constipation [27,28,29,30,31]. Conversely, some researchers suggest that constipation may be an independent risk factor for the development of depression [32]. Consequently, a review of the aforementioned literature indicates that there are many factors related to the prevalence of constipation that have not yet been sufficiently investigated, while some studies have yielded contradictory results, particularly regarding the association between depressive symptoms, DPs and constipation. There is little data about the associations between depressive symptoms, DPs, an individual’s sitting time (ST) and the risk of constipation. Studies on the factors associated with the incidence of constipation in Poland are very rare and usually conducted in small groups. A particularly poorly investigated issue in Poland is the associations between depressive symptoms and the prevalence of constipation. The aim of this study is to investigate factors associated with constipation, defined as a low stool frequency, in a community-dwelling middle-aged human adult population. An assumption was made that the likelihood of constipation may be increased by factors such as being of the female sex, the incidence of depressive symptoms, unhealthy DPs, a long sitting time and obesity, and that conversely, this incidence may be decreased by being of the male sex, current smoking, a long PA, normal weight, and adherence to a diet rich in fruits and vegetables.

## 2. Materials and Methods

### 2.1. Study Design and Sample Collection

**Figure 1:**
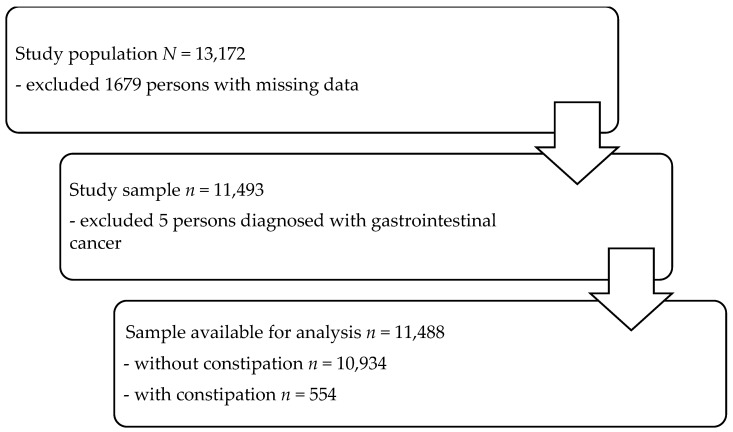
Flow diagram showing the study design and the sample.

A secondary analysis was performed with the data obtained from the PONS project (POlish–Norwegian Study), which was conducted in the Kielce District of Poland. The background of the project and the data collection method were approved by the Ethics Committee at the Cancer Centre and Institute of Oncology in Warsaw, No. 69/2009/1/2011. All procedures performed in the study followed the institution’s ethical standards, the Helsinki Declaration, and its later amendments. All the community-dwelling men and women aged from 45 to 64 years living in the Kielce District in Poland were invited to participate in the study. A detailed description of the conducted study and the recruitment method is presented in earlier publications [33,34]. The participants came from the general populations of two geographically distinct regions: one urban district (the city of Kielce) and one rural district (the district of Kielce). These regions contained a diverse but stable mix of long-term residents. The recruitment time was 16 months, and the recruitment rate was 12%. An additional, small (0.66%) group of younger (37–44 years) and older (65–66 years) participants than the initially planned age group was included in the study. As a result, a total of 13,172 persons aged 37–66 years participated in the project. Recruitment into PONS was based on voluntary participation, and the cohort was not a representative sample of the national or local population. The inclusion criteria for this analysis were permanent residency in the local population and age 37–66 years. The exclusion criteria were a diagnosis of gastrointestinal cancer at any point in time and incomplete data. After the persons with incomplete data (*n* = 1679) and persons diagnosed with gastrointestinal cancer (*n* = 5) were excluded, the analyses were conducted among a group of 11,488 persons (7633 women) (Figure 1). The Committee on Bioethics at the Faculty of Health Sciences, Jan Kochanowski University in Kielce, Poland, approved the data analysis (No. 45/2016).

### 2.2. Socio-Demographic Variables, Health and Lifestyle Data

The following sociodemographic data was collected using face-to-face interviews: sex, age, education, health and lifestyle information, and information about the occurrence of cancer in the family (in a participant’s parents or siblings). Information about stool frequency over the preceding six months was also collected. The following categories of answers to this question were used in the questionnaire: more than twice a day, twice a day, once per day, every other day, two times per week, once per week, less than once per week, I don’t know and refused to answer. A stool frequency of less than three times per week was considered to be an indicator of constipation. This problem must have been chronic during this period. Symptoms of depression were assessed based on a score obtained for answers to eight questions about a participant’s well-being over the preceding 12 months (where ‘yes’ = 1 point; ‘no’ = 0 points): fatigue (loss of energy), trouble concentrating, trouble sleeping, sadness (distress), loss of interest and feelings of pleasure, weight gain or weight loss, feeling helpless (low self-esteem) and thoughts of death. The participants were divided into three tertile groups: persons with severe (6–8 points), average (3–5 points), and mild or no depressive symptoms (0–2 points). Information about regular use of antidepressants over the preceding 30 days was also collected (‘yes’ or ‘no’).

The lifestyle assessment included eating frequency, PA and smoking. The semi-quantitative Food Frequency Questionnaire (FFQ) used in the study was constructed based on a previously developed and validated FFQ for the Poland branch of the PURE (Prospective Urban and Rural Epidemiological) study. Research has confirmed its good degree of accuracy and repeatability compared to a reference method [35]. The FFQ provided a list of 67 standard-sized products. The participants selected how often they ate a portion of each product over the preceding year from the following categories of answers: ‘6 times per day or more’, ‘4–5 times per day’, ‘2–3 times per day’, ‘once per day’, ‘5–6 times per week’, ‘2–4 times per week’, ‘once per week’, ‘1–3 times per month’, ‘less than once per month or not at all’, ‘I don’t know’ and ‘I refuse to answer’. In order to identify the DPs, the food products and drinks from the questionnaire were combined into 33 groups of foods with similar nutritional value. The group of current smokers included participants who smoked every day, whereas the group of past smokers included those who, at the time of the study, had not smoked for more than six months. The remaining participants were classified as non-smokers. Physical activity was assessed using the long version of the International Physical Activity Questionnaire (IPAQ). The moderate-to-vigorous PA (MVPA) time and sitting time (ST) were calculated in terms of minutes per week.

### 2.3. Anthropometric Measurements

Height was measured using a stadiometer, while body mass was measured using a body composition analyser (Tanita S.C. 240 MA, Tokyo, Japan). The measurement accuracies amounted to 0.1 cm and 0.1 kg, respectively. These measurements were used to calculate the BMI (kg/m^2^).

### 2.4. Statistical Analyses

#### 2.4.1. Identification of DPs

Dietary patterns were identified using a factor analysis. The responses from the FFQ concerning the frequency of the consumption of specific food products were transformed into daily doses and were normalised using the *Z*-score procedure. The conducted tests, i.e., the Kaiser–Meyer–Olkin test (0.661) and Bartlett’s test of sphericity (*p* < 0.001), confirmed the validity of conducting a factor analysis due to the fairly strong associations found between the factors. The factor analysis excluded the products that showed no relationship or a weak relationship with other food products. Thus, oils and other vegetable fats, tea, soft drinks and fruit juices, animal fats, fish, hard cheeses, mayonnaise, French fries and dumplings were not included in the factor analysis. The list of factors was created based on a scree plot (Cattell criterion) and the Kaiser criterion (> 1). The normalised Varimax procedure was used for a factor rotation. For the food products that remained in the equations, a factor loading of ≥0.35 was considered significant. The conducted analyses indicated four dietary factors that together explained 34.5% of the overall variation.

#### 2.4.2. Statistical Processing of the Results

For all categorised variables (sex, education, smoking, stool frequency, depressive symptoms, use of antidepressants, gastrointestinal cancers in the family and BMI), the totals and percentages were calculated for the whole group. For variables with a continuous distribution, means and standard deviations (age), as well as medians and the interquartile range (MVPA and ST), were calculated after testing the normalcy of distributions using the Kolmogorov–Smirnov test with the Lilliefors correction. Due to the reliability of the data, we decided not to complement the missing responses and information in a statistical manner. Centile values were counted within each dietary factor as 33.33 and 66.67, based on which all the factor values were categorised into the individual tertiles (T1–T3) of the separated DPs. The relationship between a DP and the likelihood of constipation was assessed using a logistic regression analysis, and the OR and 95% CI were calculated. The reference groups were as follows for all DPs: the first, lowest tertile (T1), normal stool frequency (≥3 times per week) and mild or no depressive symptoms (0–2 points). Each model also included the following associated variables: sex (ref.: male), education (ref.: primary), BMI (ref.: <25 kg/m^2^), smoking (ref.: non-smoker), MVPA (ref.: Q1—lowest), ST (ref.: Q1—lowest) and the use of antidepressants (ref.: no). In addition, the models were corrected with the following variables: age (as a continuous variable), education (ref.: primary) and gastrointestinal cancer diagnosed in a participant’s family at any point (ref.: no). The variables were included in the model based on analyses of the relationships between the categorised variables (chi-square) and continuous variables (correlation matrices). The basis for their inclusion in the analysis was a significant value of the correlation coefficient between the dependent variable and the independent variable, and a weak or insignificant relationship between the independent variables. Significance was assumed at *p* < 0.05. The statistical analysis was conducted using the Statistical Package STATISTICA 13.3 software.

## 3. Results

The analysed group consisted of 7633 (66.4%) women and 3855 (33.6%) men, with a mean age of 55.61 ± 5.38 years (Table 1). Over 70% of the participants had completed at least secondary education. A large percentage of the participants were overweight (43.64%) or obese (30.21%). Constipation (stool frequency <3 times per week) was declared by 554 persons (4.82%). Over 25.3% of the participants showed moderate depressive symptoms and 11.5% showed severe symptoms. Only 2.86% used antidepressants regularly. Almost 13% of the participants declared the incidence of gastrointestinal cancer in their closest families. Non-smokers made up 46.74% and past smokers made up 33.81% of the analysed group. The median of the MVPA time was 360 min/week, while the median of the ST was 1800 min/week.

A factor analysis was used to distinguish four DPs: ‘Carbohydrates’, ‘Vegetables and Meat’, ‘Healthy’ and ‘Unhealthy’. DP I—‘Carbohydrates’—showed a positive correlation with the intake of white bread and other refined flour products, sweets, potatoes, soups and processed meat, while showing a negative correlation with the intake of whole-grain cereal products (Table 2). DP II—‘Vegetables and Meat’—correlated with the intake of vegetables, sauerkraut, vegetable salad made of cooked vegetables, red meat and poultry, bigos (a stew made of sauerkraut, meat and mushrooms), eggs and mayonnaise. DP III—‘Healthy’—showed the strongest positive correlation with the intake of fruits, vegetables, low-fat dairy products and whole-grain cereal products, while showing a negative correlation with the intake of white bread and other products made from refined flour. Finally, DP IV—‘Unhealthy’—showed a positive correlation with the intake of alcohol, sweetened soft drinks, coffee, processed meat and eggs.

Women showed more frequent constipation, moderate and severe depressive symptoms and use of antidepressants, and slightly higher PA and a Healthy DP, compared to men (Table S1). Conversely, men smoked, were overweight or obese, and their diet belonged to T3 of the Unhealthy, Carbohydrates or Vegetables and Meat DP more often than women. Participants who used antidepressants within the preceding 30 days presented with constipation and smoked more often and used the Unhealthy DP less often (Table S2). The likelihood of constipation was significantly increased by the occurrence of moderate and severe depressive symptoms (compared to mild or no symptoms) (all *p* < 0.001), as well as the use of antidepressants, the longest ST (Q3 and Q4 compared to Q1) and past smoking (Figure S1). Conversely, the likelihood of constipation in each DP was significantly decreased by male sex (all *p* < 0.001), obesity (compared to BMI < 25.0 kg/m^2^) and a diet belonging to the T3 of the ‘Carbohydrates’, ‘Vegetables and Meat’ and ‘Healthy’ DPs, as compared to T1 (Table 3). In the ‘Unhealthy’ DP, the likelihood of constipation was additionally significantly decreased by a high PA (Q4 vs. Q1 of MVPA).

An analysis conducted separately for both genders showed that in both men and women, a higher likelihood of constipation was associated in each DP with moderate and severe depressive symptoms (compared to mild or no symptoms), use of antidepressants and the longest sitting time (Q4 vs. Q1) (Figures S2–S9). In addition, constipation in women was associated with past smoking. A lower likelihood of constipation in women was significantly associated with obesity (compared to BMI < 25.0 kg/m^2^) and a diet belonging to T3 of the Carbohydrates (Figure S2) and Carbohydrates and Vegetables (Figure S4) DPs, or a diet belonging to T2 and T3 of the Healthy DP (Figure S6), as compared to T1. In men, a lower likelihood of constipation was associated with a diet belonging to T2 and T3 of the Carbohydrates and Vegetables DP (Figure S5).

## 4. Discussion

According to our knowledge, this is the first study conducted in Poland that assessed the prevalence of constipation and its associations with DPs and depressive symptoms. The likelihood of constipation (defined as an abnormally low stool frequency) was significantly increased by moderate and severe depressive symptoms and the regular use of antidepressants. The results of the conducted analyses are consistent with the results of cross-sectional studies, performed in the US, the UK, Finland, Iran and Japan, which demonstrated that persons with a diagnosis of depression declared constipation much more frequently than persons without a diagnosis of depression [28,29,30,31]. This relationship was observed independently from the severity of the depression [36]. Constipation can also be a side effect of depression treatment [37]. However, some researchers have suggested that, on the contrary, constipation may be an independent risk factor of depression [32]. A reverse cause-and-effect assessment, confirmed by a bidirectional Mendelian randomization analysis, showed a causal relationship between depression and constipation. Furthermore, no evidence was found for the reverse relationship (i.e., constipation increasing the risk of depression) [30,31,38]. Depression may lead to constipation through a variety of interconnected mechanisms, including an interaction between the central nervous system and the digestive tract and changes in an individual’s lifestyle and diet. Research indicates that the corticotropin-releasing hormone in the hypothalamus is an important regulator of the relationship between emotional stress and changes in the motor functions of the digestive tract, and it may be involved in the development of stress-related gastrointestinal disorders, such as chronic constipation [31,39]. A low serotonin concentration, which may be both a symptom of depression [40] and a cause of slow peristalsis, is also related to both problems [41,42]. A recent study demonstrated that dysbiosis of the gut microbiota may lead to an abnormal production of neurotransmitters, metabolites and other substances that can negatively affect both a person’s mood and brain functions, as well as the occurrence of constipation [43]. Furthermore, persons with depression may modify their dietary habits (e.g., reduce their overall food intake or increase the intake of unhealthy food) and have a reduced PA. The research conducted to date has indicated that a low PA correlates significantly with an increased frequency of constipation [7]. However, many researchers treat ST as a separate factor that acts independently from PA. The available literature contains few works concerning the direct effect of ST on the risk of constipation. Xiong et al. [44] reported that persons with a higher ST who also slept for less than 7 h per day showed a higher risk of constipation than persons with a shorter ST who slept for a longer time. A sedentary lifestyle may disrupt the activity of the autonomous nervous system, contributing to the development of constipation by reducing the activity of the parasympathetic nervous system, which is necessary for proper bowel functions. This study confirmed that a long ST (≥1800 min/week compared to <540 min/week) increased the likelihood of constipation. Current smoking did not correlate significantly with the risk of constipation, whereas past smoking increased the risk by over 40% in women. These results are consistent with those obtained by some authors, who have suggested that quitting smoking, despite the undeniable long-term health benefits, may be associated with constipation as a harmful, short-term physiological effect [20,21,22]. However, the hypothesis that current smoking is associated with a lower likelihood of constipation has not been proven. This may be due to interactions with other factors, such as symptoms of depression, as some long-term studies have indicated that cigarette smokers present with depression much more frequently than non-smokers [45]. The results of this study, which indicate that men experience constipation less frequently than women, are consistent with almost all the epidemiological studies and literature reviews [7]. This is related to the fact that female sexual hormones may affect peristalsis, and that a decrease in these hormones, especially during menopause, may lead to constipation [17,46]. However, the main factors associated with a higher likelihood of constipation were the same in both genders. Diet is one of the most important factors affecting peristalsis and stool frequency [47]. Moreover, people with functional gastrointestinal disorders may have specific DPs that affect the frequency and severity of gastrointestinal symptoms [48]. The analyses conducted in this study demonstrate that persons whose diets belong to the third, highest, tercile of the factor values of the ‘Carbohydrates’, ‘Vegetables and Meat’ and ‘Healthy’ DPs show a significantly lower likelihood of constipation compared to persons from the T1 of the same DPs. The ‘Unhealthy’ DP was the only pattern in which an analogous relationship was not observed. However, the DPs associated with a lower likelihood of constipation differed slightly between genders. Constipation occurred the least often in men who followed the Vegetables and Meat DP and in women who followed the Healthy DP. To date, few studies have been conducted concerning the relationship between DPs and the risk of constipation. A decreased risk of constipation was noted in persons from the highest traditional southern Chinese quartile DP [13]. In Iran, a significantly lower risk of constipation was observed in persons who were recorded as less than median in the western DP, whereas a diet poor in fruits and vegetables increased this risk [14]. A prospective analysis of DPs conducted in the US confirmed that a long-term Western diet increased the risk of constipation, whereas following a Mediterranean diet rich in vegetables decreased it [16]. The fact that in the group analysed in this study, the highest tertiles of the ‘Healthy’ and ‘Vegetables and Meat’ DPs, which correlated primarily with a high vegetable intake, were the least likely to be reported alongside constipation, is consistent with the results obtained in most of the studies conducted to date [9,11,14,17,49]. Conversely, the lower risk of constipation in the third tertile (T3) of the ‘Carbohydrates’ DP may have resulted from the higher intake of starch and simple sugars, which may even cause diarrhoea by stimulating fermentation and contributing to the accumulation of water in the gut [50].

The results of studies conducted on the relationship between obesity and constipation are ambiguous [7]. A reverse relationship between obesity and the risk of constipation was found in a Peruvian population [5]. Another study in a large French adult population did not find any significant links between obesity and the prevalence of constipation [24]. Our study did not confirm the hypothesis that obesity is associated with an increased risk of constipation. The analysed group showed an over 30% lower risk of constipation in women with obesity compared to women with a BMI < 25 m/kg^2^. These findings are consistent with the results obtained in many earlier publications conducted in the US, Iran, China and Japan [25,26,51,52], even though, according to some of these studies, the associations may differ depending on the participants’ sex [52]. The lower likelihood of constipation in persons with obesity may result from a higher overall intake of food including nutrients such as fibre, which reduces the risk of constipation [11], as well as from a high intake of fermenting sugars, which increase the frequency of diarrhoea [50].

The results of this study show a lack of significant relationships between PA and constipation, with one exception: in the ‘Unhealthy’ DP, a high PA (≥Q3 of MVPA) reduced the likelihood of abnormally low stool frequency by 25%. The results obtained by other authors have demonstrated a reduced risk of constipation in physically active persons [7]. This is because PA and the hormonal changes that occur during exercise may have a positive effect on the digestive tract [53]. However, analyses conducted based on data from the Finnish database (FinnGen) did not confirm the existence of a relationship between PA and constipation [54]. Such a relationship may therefore be caused by differences in the responses of individuals’ digestive tracts to exercise and specific components of their diet and by interactions with other factors, including factors that this study did not take into account.

### Limitations

The limitations of this study were primarily related to its cross-sectional design and it only being possible to analyse a single indicator of constipation (stool frequency), rather than a full medical diagnosis of constipation. However, stool frequency is largely correlated with the stool texture [55]. Furthermore, because the study was based exclusively on data from volunteers, the analysed group does not accurately represent the entire population, which limits the generalisability of the obtained results to the entire population. The study was also not designed to include the potential use of laxatives and other drugs that may affect stool frequency, such as opioid analgesics. The difficulty of accurately estimating portion sizes among respondents should also be considered. Moreover, the nutrient content (including dietary fibre) of the same foods and dishes consumed by different people can vary significantly. Conversely, a strength of this study was the large sample size and relatively narrow age range, and the large number of associated variables that were included in the analyses.

## 5. Conclusions

The factors associated with constipation (low stool frequency) were found to be moderate and severe depressive symptoms, regular use of antidepressants, a sedentary lifestyle and past smoking (in women). In turn, the factors associated with a lower likelihood of constipation were a diet belonging to the highest terciles of the ‘Carbohydrates’, ‘Vegetables and Meat’ and ‘Healthy’ DPs, a male sex and obesity in women. In the case of the ‘Unhealthy’ DP, the likelihood of constipation was decreased by a high PA.

The risk of constipation should be decreased by preventing the onset or reoccurrence of depression, i.e., by improving peoples’ mental resistance, reducing the risk factors of depression and promoting a healthy lifestyle, primarily a diet high in vegetables and a limited sitting time. Further longitudinal and randomised control studies are needed to better understand the factors that affect the incidence of constipation and the interactions between them.

## Figures and Tables

**Table 1:** Characteristics of the sample (*N* = 11,488).

Variable	Category	*N*	%
Sex	Men	3855	33.56
	Women	7633	66.44
Age (years)	*X* ± *SD*	55.61 ± 5.38	
Education	Primary	997	8.68
	Vocational	2354	20.49
	Secondary	4702	40.93
	Bachelor’s degree	428	3.73
	Master’s degree	3007	26.17
BMI (kg/m^2^)	<25.0	3004	26.15
	25.0–29.9	5013	43.64
	≥30.0	3471	30.21
Stool frequency over the last six months	≥3 times per week	10,934	95.18
	<3 times per week	554	4.82
Depressive symptoms (points)	Mild or none (0–2)	7263	63.22
	Moderate (3–5)	2903	25.27
	Severe (6–8)	1322	11.51
Use of antidepressants	Yes	329	2.86
	No	11,159	97.14
Gastrointestinal cancer in the family	Yes	1477	12.86
	No	10,011	87.14
Smoking	Non-smoker	5369	46.74
	Past smoker	3884	33.81
	Current smoker	2235	19.46
MVPA (min/week)	*Me* (IQR)	360.0 (570.0)	
Sitting time (min/week)	*Me* (IQR)	1800.0 (1260.0)	

*X*—arithmetic mean; *SD*—standard deviation; *Me*—median; IQR—interquartile range; BMI—Body Mass Index; MVPA—Moderate-to-Vigorous Physical Activity.

**Table 2:** Factor-loading matrix for major dietary patterns *.

Food Group	Factor			
	I	II	III	IV
Fruits			0.629	
Vegetables		0.431	0.517	
Milk and yoghurt			0.589	
Curd cheese			0.499	
Whole grains	−0.452		0.564	
Refined grains	0.630		−0.413	
Potatoes	0.675			
Soups	0.557			
Vegetable salad		0.399		
Sauerkraut		0.585		
Bigos		0.688		
Red meat		0.450		
Processed meat	0.351			0.362
Poultry		0.454		
Eggs				0.450
Alcohol				0.524
Coffee				0.492
Sweetened soft drinks				0.495
Sugar and sweets	0.377			
Percentage of variance explained (%)	17.05	16.77	19.41	12.31

* Values < 0.35 were excluded.

**Table 3:** Factors associated with constipation (low stool frequency).

Factor	Category	I. Carbohydrates		II. Vegetables and Meat		III. Healthy		IV. Unhealthy	
		OR (95%CI)	*p*	OR (95%CI)	*p*	OR (95%CI)	*p*	OR (95%CI)	*p*
Gender Ref.: women	Men	0.15; 0.10–0.20	<0.001	0.14; 0.10–0.20	<0.001	0.13; 0.10–0.19	<0.001	0.14; 0.10–0.20	<0.001
DP Ref.: T1	T2	0.84; 0.68–1.13	0.294	0.89; 0.73–1.09	0.263	0.83; 0.67–1.03	0.094	1.19; 0.96–1.47	0.109
	T3	0.76; 0.61–0.95	0.018	0.72; 0.58–0.90	0.004	0.81; 0.65–0.99	0.049	1.12; 0.89–1.41	0.349
BMI Ref.: <25.0 kg/m^2^	Overweight	0.85; 0.69–1.05	0.294	0.87; 0.70–1.07	0.179	0.86; 0.70–1.06	0.171	0.85; 0.69–1.05	0.138
	Obesity	0.67; 0.52–0.85	0.001	0.69; 0.54–0.88	0.003	0.69; 0.54–0.88	0.002	0.68; 0.54–0.87	0.002
Depressive symptoms Ref.: 0–2 points	Moderate (3–5 points)	1.64; 1.35–1.99	<0.001	1.66; 1.36–2.02	<0.001	1.63; 1.34–1.99	<0.001	1.64; 1.35–2.00	<0.001
	Severe (6–8 points)	1.63; 1.27–2.10	<0.001	1.65; 1.28–2.12	<0.001	1.61; 1.25–2.07	<0.001	1.64; 1.27–2.11	<0.001
Antidepressants Ref.: no	Yes	1.74; 1.19–2.54	0.004	1.71; 1.17–2.49	0.006	1.75; 1.20–2.55	0.004	1.75; 1.20–2.55	0.004
Sitting time Ref.: Q1	Q2	1.04; 0.78–1.37	0.799	1.03; 0.78–1.36	0.828	1.02; 0.77–1.35	0.876	1.03; 0.78–1.36	0.842
	Q3	1.36; 1.04–1.78	0.026	1.34; 1.03–1.76	0.032	1.33; 1.02–1.74	0.038	1.35; 1.03–1.77	0.029
	Q4	1.54; 1.18–2.01	0.002	1.51; 1.16–1.97	0.002	1.49; 1.14–1.95	0.003	1.53; 1.17–1.99	0.002
MVPA Ref.: Q1	Q2	0.98; 0.76–1.27	0.880	0.98 0.76–1.26	0.845	0.98; 0.76–1.27	0.902	0.86; 0.76–1.26	0.845
	Q3	0.95; 0.74–1.22	0.667	0.93; 0.73–1.21	0.615	0.94; 0.73–1.21	0.626	0.93; 0.72–1.19	0.569
	Q4	0.78; 0.59–1.03	0.080	0.78; 0.59–1.03	0.083	0.77; 0.58–1.02	0.064	0.75; 0.57–0.99	0.048
Smoking Ref.: non-smoker	Past smoker	1.43; 1.18–1.74	<0.001	1.45; 1.20–1.76	<0.001	1.46; 1.21–1.78	<0.001	1.43; 1.18–1.74	<0.001
	Current smoker	0.87; 0.68–1.13	0.294	0.86; 0.67–1.11	0.237	0.85; 0.66–1.10	0.216	0.84; 0.65–1.09	0.184

The model was adjusted for age, education and occurrence of gastrointestinal cancer in a participant’s family. Ref.—reference value; Q—quartile; OR—odds ratio; CI—confidence interval; DP—dietary pattern; BMI—Body Mass Index; MVPA—Moderate-to-Vigorous Physical Activity.

## Data Availability

The datasets used and/or analysed during the current study are available from the corresponding author upon reasonable request.

## Supplementary Materials

The following supporting information can be downloaded at: https://www.mdpi.com/article/10.3390/jcm14197090/s1, Table S1. Characteristics of the sample depending on sex; Table S2. Characteristics of the sample depending on the use of antidepressants; Figure S1. Multiple logistic regression analyses with the risk factors for the occurrence of constipation (total); Figure S2. Multiple logistic regression analyses with the risk factors for the occurrence of constipation in women in DP I—Carbohydrates; Figure S3. Multiple logistic regression analyses with the risk factors for the occurrence of constipation in men in DP I—Carbohydrates; Figure S4. Multiple logistic regression analyses with the risk factors for the occurrence of constipation in women in DP II— Vegetables and Meat; Figure S5. Multiple logistic regression analyses with the risk factors for the occurrence of constipation in men in DP II—Vegetables and Meat; Figure S6. Multiple logistic regression analyses with the risk factors for the occurrence of constipation in women in DP III—Healthy; Figure S7. Multiple logistic regression analyses with the risk factors for the occurrence of constipation in men in DP III—Healthy; Figure S8. Multiple logistic regression analyses with the risk factors for the occurrence of constipation in women in DP IV—Unhealthy; Figure S9. Multiple logistic regression analyses with the risk factors for the occurrence of constipation in men in DP IV—Unhealthy.

## Abbreviations

The following abbreviations are used in this manuscript:

DP	Dietary pattern
Q	Quartile
MVPA	Moderate-to-Vigorous Physical Activity
OR	Odds Ratio
PONS	POlish–Norwegian Study
BMI	Body Mass Index
FFQ	Food Frequency Questionnaire
PURE	Prospective Urban and Rural Epidemiological study
IPAQ	International Physical Activity Questionnaire
ST	Sitting time
CI	Confidence Interval
T	Tertile
IQR	Interquartile range
DS	Depressive Symptoms
