# Supplementary material for: Depressive Symptoms, Dietary Patterns and Other Factors Associated with Constipation in Middle-Aged Adults from Kielce District in Poland: A Cross-Sectional Study
Source: J Clin Med. 2025 Oct 8;14(19):7090. doi: 10.3390/jcm14197090 (PMC12525617; doi:10.3390/jcm14197090)
Supplement: Supplementary file 1 [file jcm-14-07090-s001.zip › jcm-3814553-supplementary.pdf]

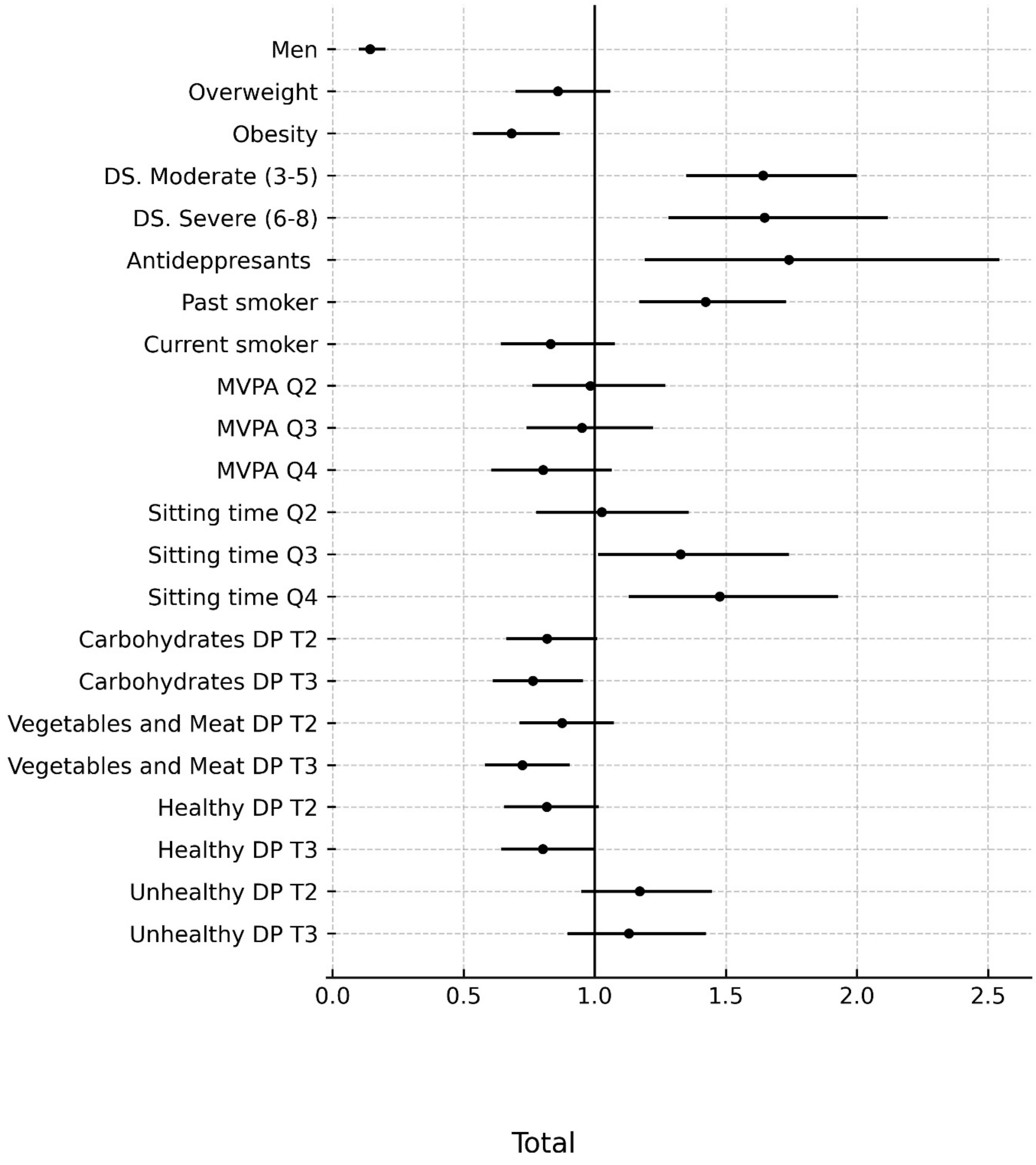

Figure S1. Multiple logistic regression analyses with the risk factors for the occurrence of constipation (total).

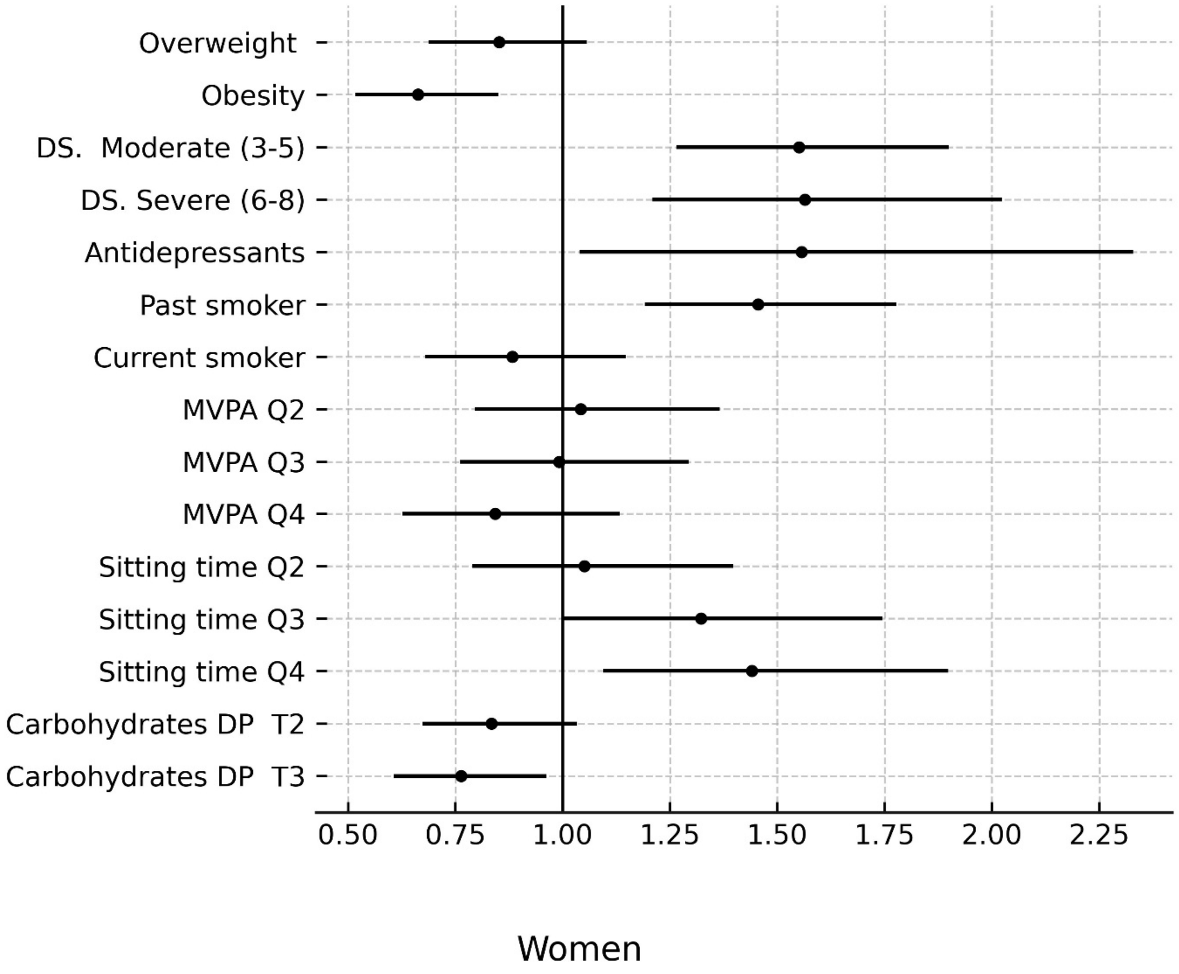

Figure S2. Multiple logistic regression analyses with the risk factors for the occurrence of constipation in women in DP I – Carbohydrates.

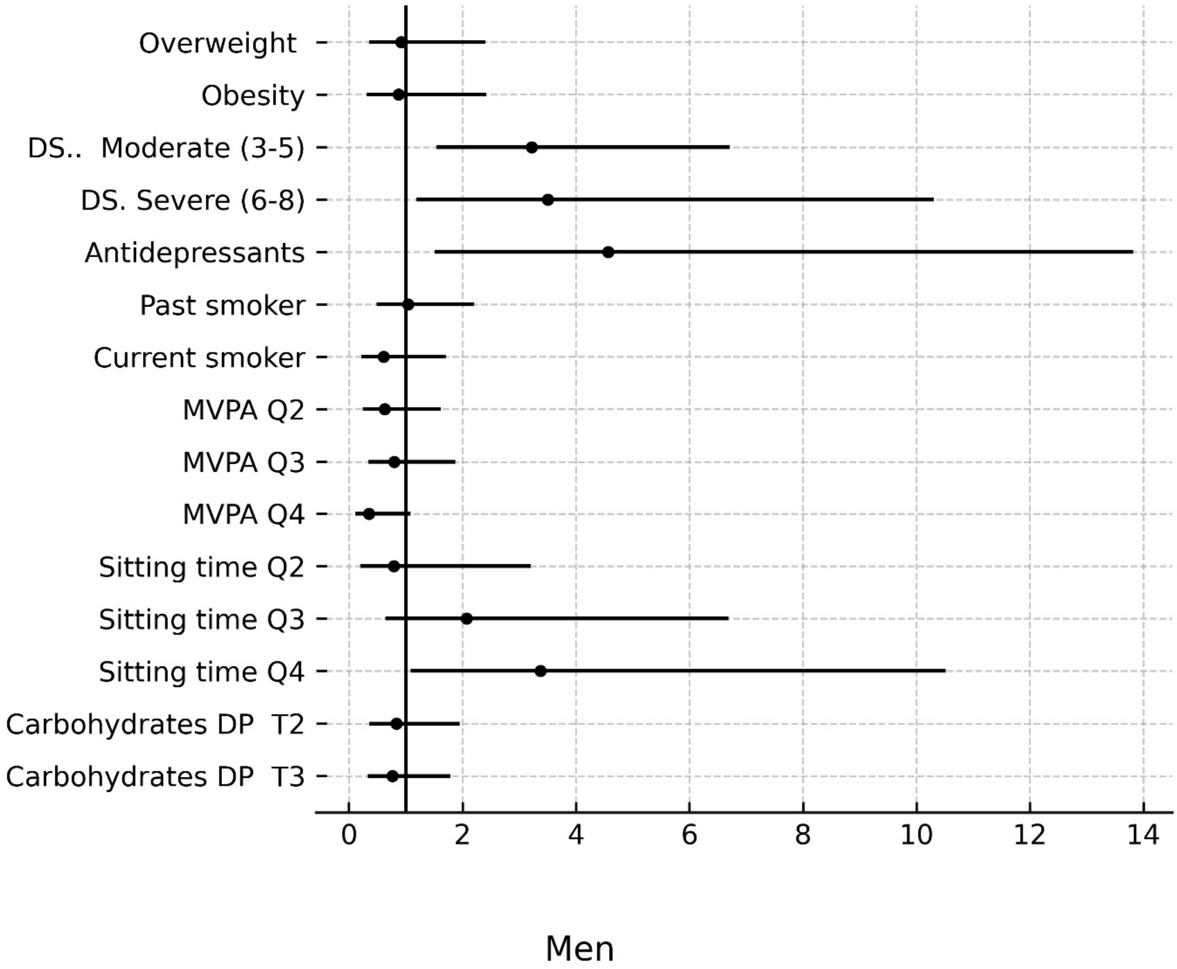

Figure S3. Multiple logistic regression analyses with the risk factors for the occurrence of constipation in men in DP I – Carbohydrates.

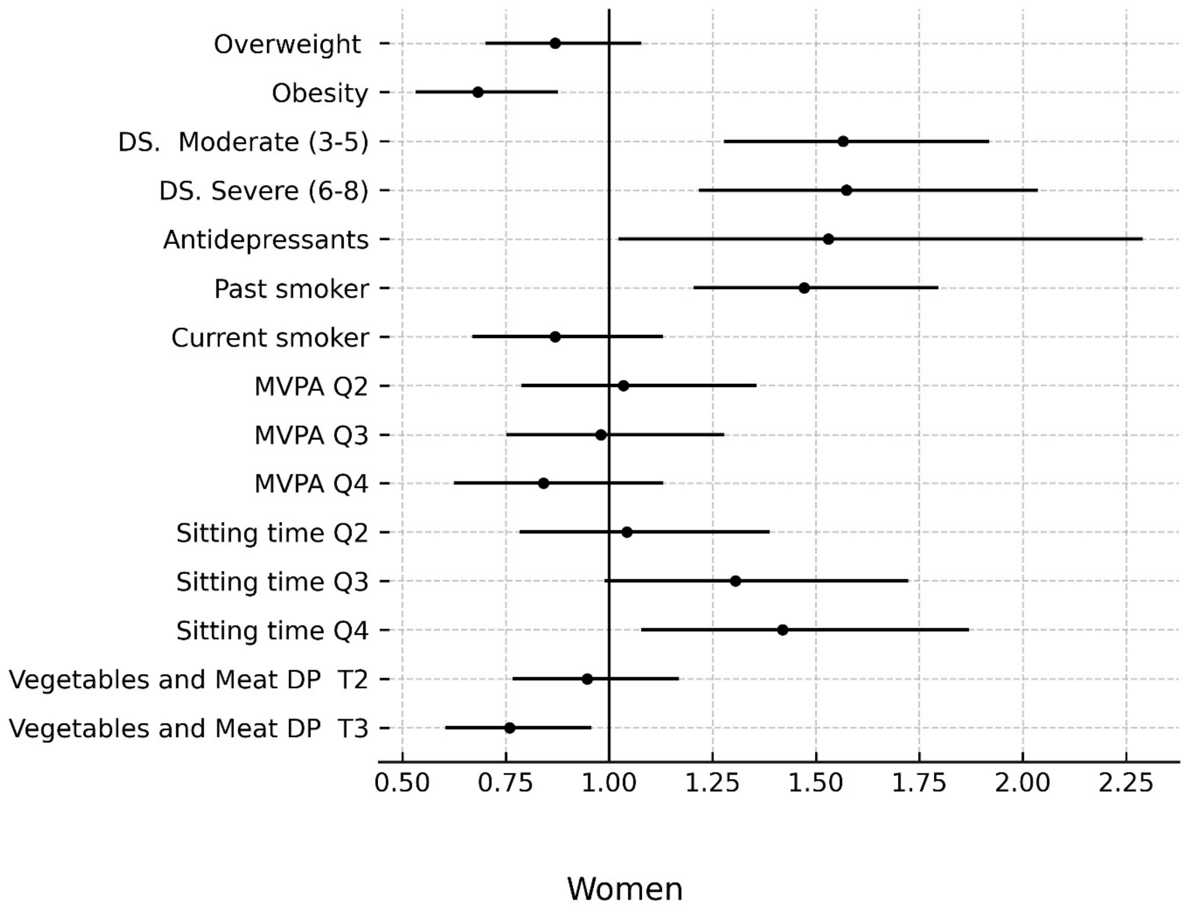

Figure S4. Multiple logistic regression analyses with the risk factors for the occurrence of constipation in women in DP II - Vegetables and Meat.

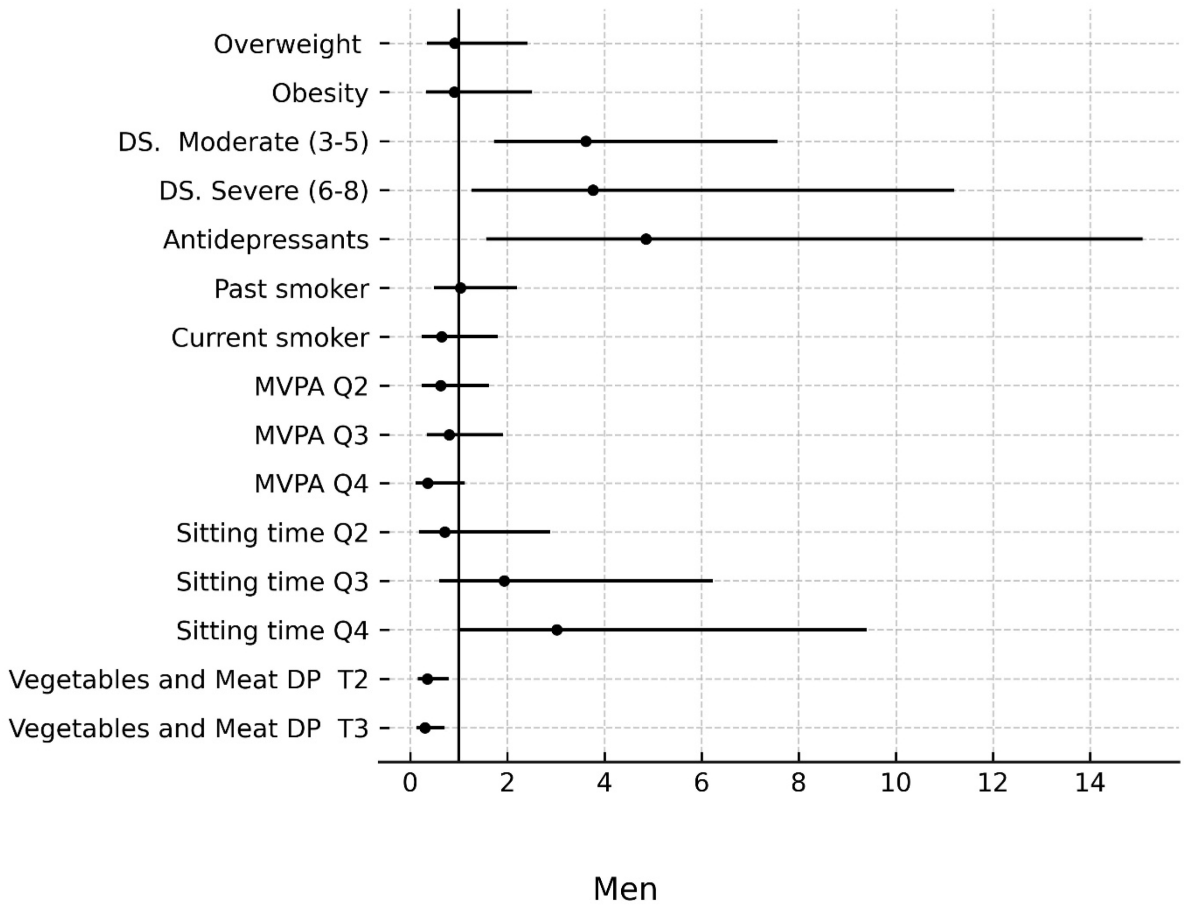

Figure S5. Multiple logistic regression analyses with the risk factors for the occurrence of constipation in men in DP II - Vegetables and Meat.

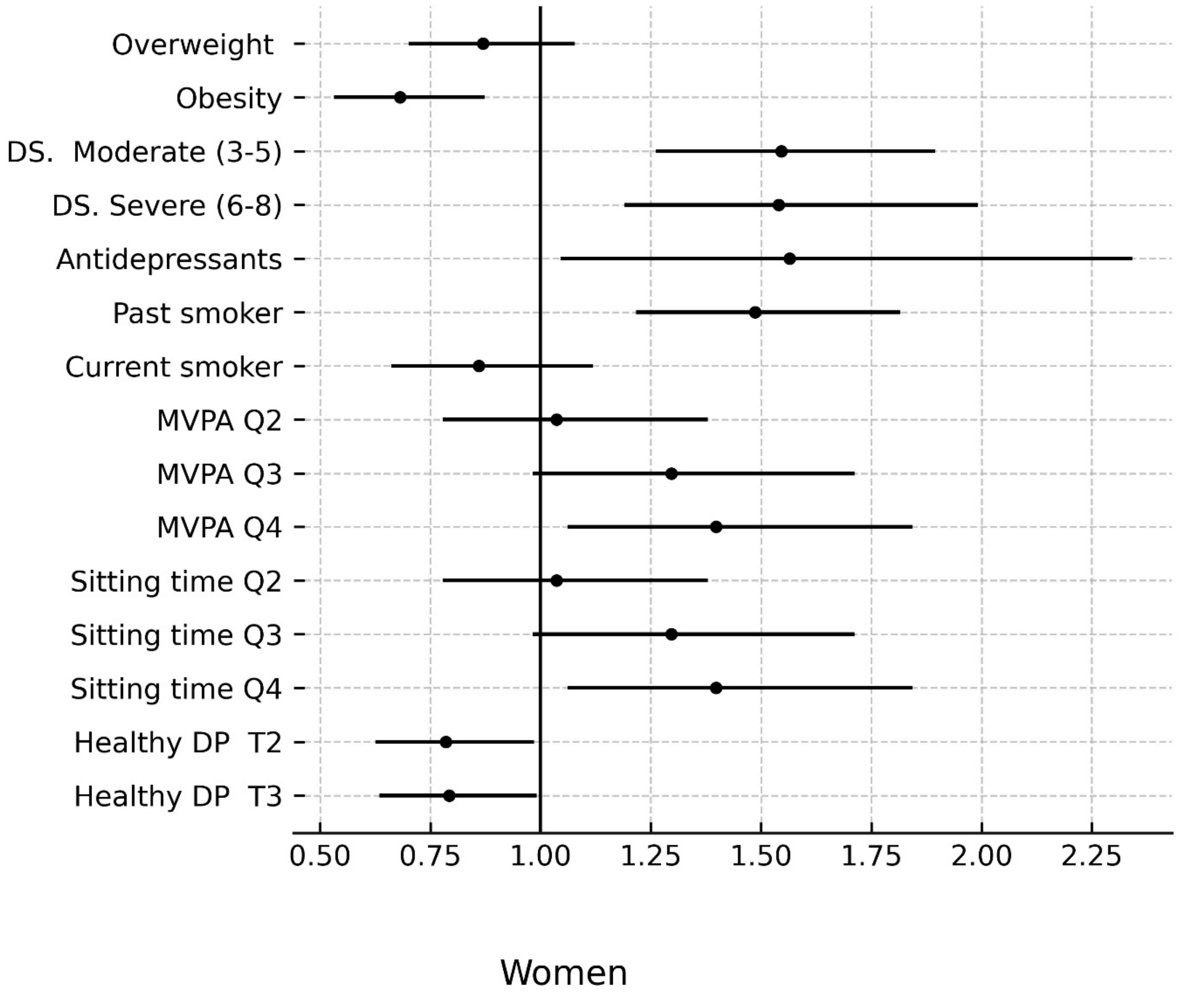

Figure S6. Multiple logistic regression analyses with the risk factors for the occurrence of constipation in women in DP III – Healthy.

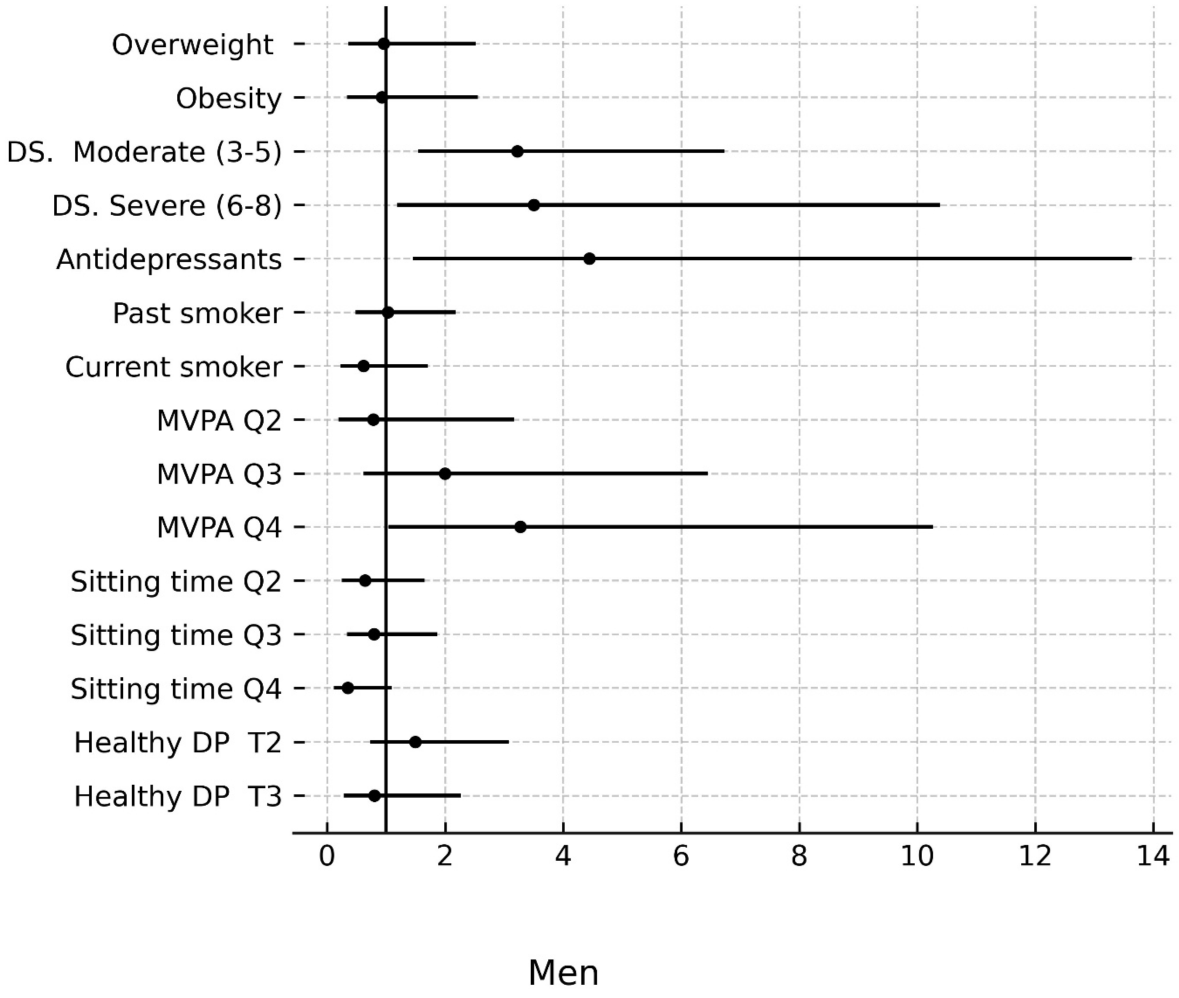

Figure S7. Multiple logistic regression analyses with the risk factors for the occurrence of constipation in men in DP III – Healthy.

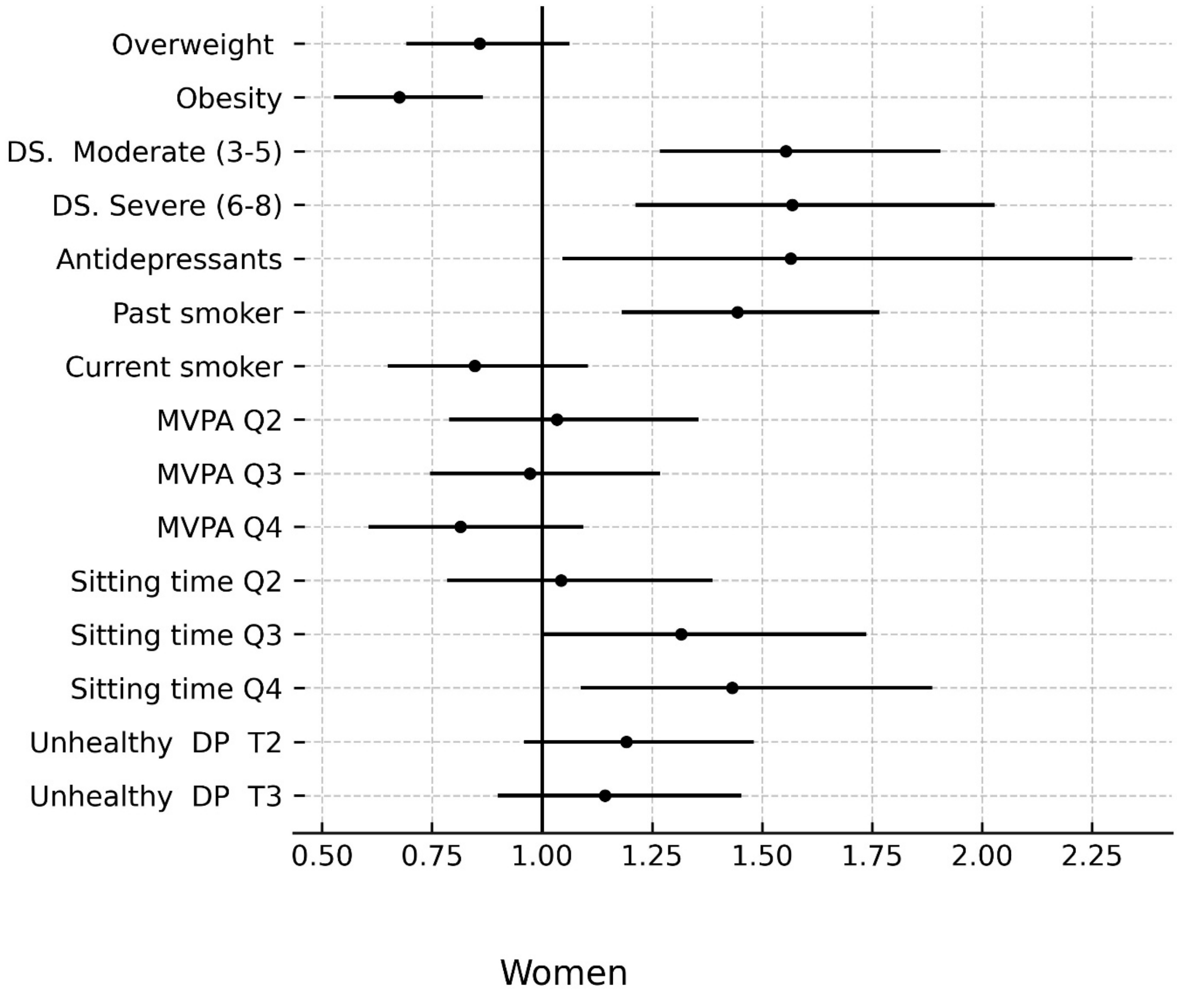

Figure S8. Multiple logistic regression analyses with the risk factors for the occurrence of constipation in women in DP IV – Unhealthy.

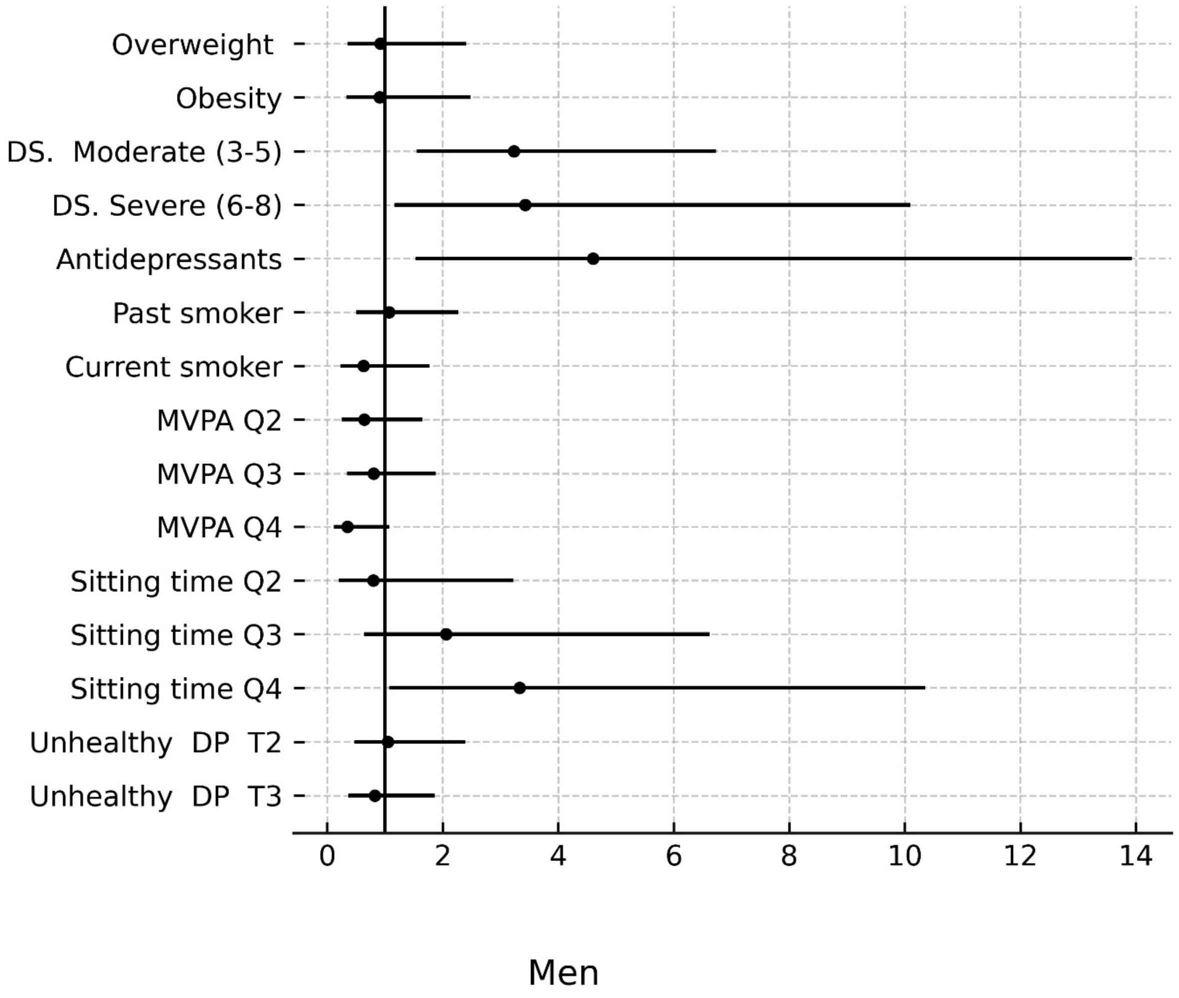

Figure S9. Multiple logistic regression analyses with the risk factors for the occurrence of constipation in men in DP IV - Unhealthy.

Table S1. Characteristics of the sample depending of sex.

Variable	Category	Women N = 7633		Men N = 3855		p
		N	%	N	%	
Age (years)	*X* ± *SD*	55.47 ± 5.36		55.89 ± 5.42		<0.001[a]
Education	Primary	733	9.60	264	6.85	<0.001[c]
	Vocational	1219	15.97	1135	29.44	
	Secondary	3304	43.29	1398	36.26	
	Bachelor's degree	308	4.04	120	3.11	
	Master's degree	2069	27.11	938	24.33	
BMI (kg/m^2)	< 25.0	2301	30.15	703	18.24	<0.001[c]
	25.0–29.9	3064	40.14	1949	50.56	
	≥ 30.0	2268	29.71	1203	31.21	
Stool frequency over the last six months	≥ 3 times per week	7116	93.23	3818	99.04	<0.001[c]
	< 3 times per week	517	6.77	37	0.96	
Depressive symptoms (points)	Mild or none (0–2)	4415	57.84	2848	73.88	<0.001[c]
	Moderate (3–5)	2120	27.77	783	20.31	
	Severe (6–8)	1098	14.38	224	5.81	
Use of antidepressants	Yes	259	3.39	70	1.82	<0.001[c]
	No	7374	96.61	3785	98.18	
Gastrointestinal cancer in the family	Yes	1053	13.80	424	11.00	<0.001[c]
	No	6580	86.20	3431	89.00	
Smoking	Non-smoker	3997	52.36	1372	35.59	<0.001[c]
	Past smoker	2227	29.18	1657	42.98	
	Current smoker	1409	18.46	826	21.43	
MVPA (min/week)	*Me* (IQR)	370.60 (555.0)		300.0 (690.0)		<0.001[b]
Sitting time (min/week)	*Me* (IQR)	1800.0 (1260.0)		1860.0 (1260.0)		0.439[b]
DP I	*T1*	2846	37.29	1000	25.94	<0.001[c]
	T2	2554	33.46	1260	32.68	
	T3	2233	29.25	1595	41.37	
DP II	*T1*	2658	34.82	1172	30.40	<0.001[c]
	T2	2599	34.05	1290	33.46	
	T3	2376	31.13	1393	36.13	
DP III	*T1*	2162	28.32	1644	42.65	<0.001[c]
	T2	2529	33.13	1285	33.33	
	T3	2942	38.54	926	24.02	
DP IV	*T1*	2715	35.57	1083	28.09	<0.001[c]
	T2	2760	36.16	1086	28.17	
	T3	2158	28.27	1686	43.74	

[a] – t-Student Test for independent variables; [b] - U Mann-Whitney test; [c] - Chi square test; DP – Dietary Pattern; BMI – Body Mass Index; MVPA – Moderate-to-Vigorous Physical Activity.

Table S2. Characteristics of the sample depending of the use of antidepressants.

**Variable**	**Category**	**Use of antidepressants**				**p**
		Yes		No		
		N	%	N	%	
Sex	Women	259	78.72	7374	66.08	0.001[c]
	Men	70	21.28	3785	33.92	
Age (years)	*X* ± *SD*	56.54 ± 4.98		55.58 ± 5.39		0.001[a]
Education	Primary	43	13.07	954	8.55	0.010[c]
	Vocational	78	23.71	2276	20.40	
	Secondary	128	38.91	4574	40.99	
	Bachelor's degree	11	3.34	417	3.74	
	Master's degree	69	20.97	2938	26.33	
BMI (kg/m^2)	< 25.0	77	23.40	2927	26.23	0.219[c]
	25.0–29.9	139	42.25	4874	43.68	
	≥ 30.0	113	34.35	3358	30.09	
Stool frequency over the last six months	≥ 3 times per week	293	89.06	10641	89.06	<0.001[c]
	< 3 times per week	36	10.94	518	10.94	
Depressive symptoms (points)	Mild or none (0–2)	51	15.50	7212	64.63	<0.001[c]
	Moderate (3–5)	109	33.13	2794	25.04	
	Severe (6–8)	169	51.37	1153	10.33	
Gastrointestinal cancer in the family	Yes	52	15.81	1425	12.77	0.105[c]
	No	277	84.19	9734	87.23	
Smoking	Non-smoker	124	37.69	5245	47.00	0.003[c]
	Past smoker	125	37.99	3759	33.69	
	Current smoker	80	24.32	2155	19.31	
MVPA (min/week)	*Me* (IQR)	350.0 (490.0)		360.0 (580.0)		0.286[b]
Sitting time (min/week)	*Me* (IQR)	1800.0 (1260.0)		1800.0 (1260.0)		0.647[b]
DP I	*T1*	105	31.91	3741	33.52	0.124[c]
	T2	126	38.30	3688	33.05	
	T3	98	29.79	3730	33.43	
DP II	*T1*	117	35.56	3713	33.27	0.099[c]
	T2	122	37.08	3767	33.76	
	T3	90	27.36	3679	32.97	
DP III	*T1*	106	32.22	3700	33.16	0.615[c]
	T2	104	31.61	3710	33.25	
	T3	119	36.17	3749	33.60	
DP IV	*T1*	129	39.21	3669	32.88	0.044[c]
	T2	105	31.91	3741	33.52	
	T3	95	28.88	3749	33.60	

[a] – t-Student Test for independent variables; [b] - U Mann-Whitney test; [c] - Chi square test; DP – Dietary Pattern; BMI – Body Mass Index; MVPA – Moderate-to-Vigorous Physical Activity.
